# Maternal microbiome disturbance induces deficits in the offspring’s behaviors: a systematic review and meta-analysis

**DOI:** 10.1080/19490976.2023.2226282

**Published:** 2023-07-03

**Authors:** Lucas Hassib, Cilene Lino de Oliveira, Guilherme Araujo Rouvier, Alexandre Kanashiro, Francisco Silveira Guimarães, Frederico Rogério Ferreira

**Affiliations:** a Oswaldo Cruz Institute, Oswaldo Cruz Foundation, Rio de Janeiro, Brazil; b Department of Mental Health, School of Medicine of Ribeirão Preto, University of São Paulo, Ribeirão Preto, Brazil; c Department of Physiological Sciences, Center of Biological Sciences, Federal University of Santa Catarina, Florianópolis, Brazil; d Federal Institute of Education, Science, And Technology of Rio de Janeiro, Rio de Janeiro, Brazil; e Department of Dermatology, Medical Sciences Center, University of Wisconsin-Madison, Madison, WI, USA; f Department of Pharmacology, School of Medicine of Ribeirão Preto, University of São Paulo, Ribeirão Preto, Brazil

**Keywords:** Maternal microbiome disturbance, neurodevelopment, gut-brain axis, psychiatric disorder, sociability, autism spectrum disorder

## Abstract

Recent evidence has suggested that changes in maternal gut microbiota in early life may generate neurobiological consequences associated with psychiatric-related abnormalities. However, the number of studies on humans investigating this problem is limited, and preclinical findings sometimes conflict. Therefore, we run a meta-analysis to examine whether maternal microbiota disturbance (MMD) during neurodevelopment might affect the offspring during adulthood. We found thirteen studies, from a set of 459 records selected by strategy registered on PROSPERO (#289224), to target preclinical studies that evaluated the behavioral outcomes of the rodents generated by dams submitted to perinatal enteric microbiota perturbation. The analysis revealed a significant effect size (SMD = −0.51, 95% CI = −0.79 to −0.22, *p* < .001, T2 = 0.54, I2 = 79.85%), indicating that MMD might provoke behavioral impairments in the adult offspring. The MMD also induces a significant effect size for the reduction of the sociability behavior (SMD = −0.63, 95% CI = −1.18 to −0.07, *p* = 0.011, T2 = 0.30, I2 = 76.11%) and obsessive-compulsive-like behavior (SMD = −0.68, 95% CI = −0.01 to −1.36, *p* = 0.009, T2 = 0.25, I2 = 62.82%) parameters. The effect size was not significant or inconclusive for memory and anxiety-like behavior, or inconclusive for schizophrenia-like and depressive-like behavior. Therefore, experimental perinatal MMD is vertically transmitted to the offspring, negatively impacting behavioral parameters related to psychiatric disorders.

## GRAPHICAL ABSTRACT

**Figure:**
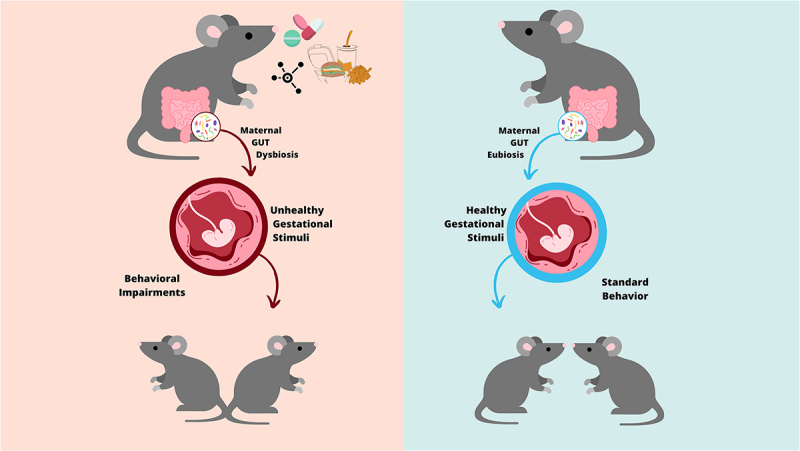

## 1. Introduction

Although the knowledge of the existence of microorganisms and their involvement in diseases begins at 15th century with the germ theory by Girolamo Fracastoro and Marcus von Plenciz, chinese medicine from the 4th century already used the Yellow Soups as a rudimentary microbiota transplantation method for treating intestinal disease.^1^ However, with recent advances in techniques for genome sequencing that allowed a deep uncovering of microbe species and variabilities,^2^ accumulated data reveal the influence of the gut microbiota on several human physiological processes, such as the sensitization and balance of the immune system^3^, hormone production,^4^ aging,^5^ and metabolism.^6^

Therefore, Sudo and colleagues^7^ provided evidence for the interplay between enteric microbiota and stress responses coordinated by the hypothalamic–pituitary–adrenal (HPA) axis. They reveal an exacerbated curve of serum glucocorticoids released after stress exposure in animal germ-free (GF) compared with the specific pathogen-free (SPF) group. Interestingly, they also highlighted that those responses of the HPA axis are normalized as GF animals are colonized with a commensal bacteria Bifidobacterium infantis. However, the regenerative effects of gut colonization are only observed as they occur in juvenile animals, before completing the neural maturation. Therefore, these findings already suggest the presence of a mechanism by which the gut microbiota could modulate the HPA stress response during neurodevelopment.^8^. Since then, the bidirectional interplay between host intestinal commensal bacteria and central nervous system (CNS) superior functions, such as emotion,^9^ effectiveness,^10^ sociability,^11^ and cognition^12^ have attracted attention. The gut-brain axis (GBA) promotes a complex bidirectional communication between gut commensal bacteria, the immune system, the enteric nervous system, and the neural system, commanding activities responsible for brain development, health, and disease.^13,14^

The role of GBA in brain development and maturation from gestational phases to adulthood neurological outcomes has been revealed in both clinical^15,16^ and preclinical studies.^17^ With different approaches, studies have highlighted the hypothesis that neurodevelopmental dysfunction induced by maternal microbiota disturbance (MMD) may result in psychiatric disorders, such as autism spectrum disorder (ASD),^18^ multiple sclerosis,^19^ generalized anxiety (GA),^20^ and schizophrenia (SZ).^21^

Although consistent results describe the neural mechanisms involving the microbiome to CNS functions, collectively, the evidence is sometimes conflicting or inconclusive.^22^ Methodological limitations of long-term follow-up of patient cohorts from pregnancy to adulthood may explain the limited number of studies and unsatisfying results.^23^ Furthermore, differences in the experimental protocols, such as the microbiota manipulation technique chosen, different behavioral analysis paradigms, and the distinct methods applied for microbiota sampling and analysis add high variability, which can result in a disagreement between preclinical studies.^24–26^ For instance, several studies using microbiota manipulation, such as diet, chemical agents, probiotics, prebiotics, or symbiotics have shown differences in the impact on bacterial populations, with divergent effects on the host outcomes.^27,28^ Therefore, we run over the current literature aimed at investigating whether MMD during the embryonic developmental window might impact the behavioral outcomes of offspring during adulthood life. To achieve this goal, we performed a systematic review with meta-analysis to combine the results of behavioral tests of the offspring generated by dams submitted to enteric microbiota perturbation during pregnancy.

## 2. Methods

### 2.1. Literature search

This study is registered to PROSPERO (#289224) and was reported according to the Preferred Reporting Items for Systematic Reviews and Meta-Analyses (PRISMA) guidelines,^29^ and the recommendations for carrying out meta-analyses.^30^ A searchable review question was structured using the PICO tool as follows: Population = laboratory rodents; Intervention = treatment of female progenitors during the pregnancy antibiotic, probiotic, prebiotic, symbiotic, microbiota colonization, microbiota transferring, and diet manipulation in any dose, via or time of administration; Control = treatment of female progenitors during the pregnancy with vehicle or saline or non-treated with intervention; Outcome = behavioral outcome of the offspring in the infancy, youth, or adulthood in relevant behavioral tests. The analyzed behaviors were those aiming to investigate depressive and anxiety-like behaviors, social deficits, cognition alterations, schizophrenic, panic, and obsessive-compulsive behaviors. A systematic computerized literature search of PubMed, EMBASE, and Web of Science was conducted to target original articles that investigate the effect of maternal microbiota perturbations on offspring behavioral outcomes using the search strategy design described in (Supplementary Table S1).

### 2.2. Eligibility criteria and screening

Rodent pre-clinical studies in any language, any date, and any journal were included if they aimed to experimentally manipulate maternal microbiota during pregnancy or until weaning and performed a behavioral analysis on offspring in adulthood. There were no studies with a protocol of MMD before gestation. The MMD window was selected due to previous clinical and preclinical evidence showing that the first offspring microbiome is mainly formed by maternal vertical transmission.^31,32^ The following inclusion criteria were applied to the screening of the relevant studies: (1) used adult laboratory rodents (rats or mice) of any sex and strain; (2) altered the maternal gestational microbiota through the use of antibiotics, probiotics, prebiotics or symbiotics, microbiota transfer, or dietary manipulations with caloric changes, fiber consumption, or ingestion of environmental contaminants; (3) characterized maternal microbiota profile; (4) the control group did not undergo any manipulation of the microbiota; (5) performed the relevant behavioral tests in the offspring older than 21 days. The following behavioral tests were eligible: Forced swimming test; Tail suspension test; Learned helplessness; Novelty suppressed feeding test; Sucrose spray test; Sucrose preference; Social default test; Elevated plus maze test; Vogel conflict test; Open field test; Light dark box; Elevated zero maze; Three chamber social interaction test; Three chamber social interaction test; Social interaction; Ultrasonic vocalization; Water maze test; Novel object recognition test; T test; Marble burying; Prepulse inhibition; Barnes maze test; and Y maze test.

Studies were excluded if they (1) were reviews, systematic reviews, or meta-analyses; (2) did not manipulate the maternal microbiota during pregnancy; (3) did not characterize the maternal microbiota; (4) performed the maternal microbiota manipulation by using behavioral or environmental stress, maternal immune-stimulating, and immune-suppressors; (5) conduct a co-treatment with two or more intervention factors; (6) performed no relevant behavioral study in the offspring of the experimental dams. Four reviewers (F.F., L.H., G.R., and A.K.) independently screened all titles and abstracts before full-text retrieval. The full texts of potentially eligible articles were assessed independently by at least two reviewers, and discrepancies were resolved through debate and consensus of the four authors for the final decision.

### 2.3. Assessment of study quality

Two independent reviewers (L.H. and G.R) evaluated the risk of bias using the RoB Syrcle tools^33^, which assess general aspects of experimental design and more specific features of animal research. In case of disagreement between the authors in some aspects of the evaluation, a third reviewer (F.F.) was consulted to make the final decision (Supplementary Table S2).

### 2.4. Data extraction, global, and stratified meta-analysis

Qualitative information was extracted by reviewers (F.F., L.H., G.R., and A.K.) using a predefined data extraction sheet (Supplementary Table S3), with the following items: article DOI, year of publication, experimental animal (species, age, and sex), microbiota manipulation technique, behavioral analysis conducted in the offspring, type of outcome measure and statistics [unity, mean, standard error, and sample size (n)]. When sample sizes were reported as intervals, the average between the numbers was used as the sample size (e.g., when 6–12 animals per group were reported, the sample size was considered as 9 animals per group).

For each primary outcome, a standardized mean difference (SMD) was calculated per study. Studies were stratified according to the theoretical paradigm (social behavior, memory, anxiety, depressive, schizophrenic, or obsessive-compulsive behavior) for subgroup meta-analysis independent of the features of the population, intervention, control, or outcomes. When feasible (at least two studies per subgroup), the meta-analyses were performed using a random effect model to estimate the combined effect size (CES, Hedges’g) with 95% confidence interval (95% CI), publication bias (Forest plot, Trim-and-Fill), and Heterogeneity (I2, Q statistic and the associated p-value, alpha = 0.05).

The combined effect size for all measures was calculated normalizing by behavioral impairment or standard behavioral, according to the outcome of the animal task. Therefore, a negative effect size was observed when treatments reduced the probability of exhibition of that particular activity (Mttd = mean of the treated group) compared to the control group (Mctl = mean of the treated group), resulting in a negative value (Mttd – Mctl < 0). For example, it was considered behavioral impairment the reduction of open arm exploration time in the Elevated Plus Maze test (interpreted as anxiety-like behavior), the reduction in the distance moved in the target quadrant in the Morris Water Maze (interpreted as memory impairment), the reduction in sugar consumption in the Sucrose Preference test (interpreted as anhedonia-like behavior), or the reduction in sociability time in the Three Chamber test (interpreted as social deficits). When the behavioral impairment results in an increase in the probability of a particular activity, such as the increase in buried marbles in the Marble Burying test (interpreted as stereotyped behavior), or increased latency to find the target hole in Barnes Maze (interpreted as memory impairments), compared to the control group, the size effect result (Mttd – Mctl) was multiplying by −1, as provided at the raw data (Supplementary table S3).

Calculations and figures were prepared using the Software Meta-essentials by Suurmond, van Rhee, and Hak^34^ (www.erim.eur.nl/research-support/meta-essentials/downloads). In the case of studies applying two or more behavioral tests to the same cohort of animals, one of them was randomly selected to be analyzed.

The combined effect sizes (CES) Hedges’g were categorized as very small (SMD = up to 0.2), small (SMD = between 0.2 and 0.5), moderate (SMD = 0.5–0.8), and large (SMD higher than 0.8).^35^ The values of I2 ranging from 0% to 100%, indicate proportion of heterogeneity interpreted as low to up to 25%, moderate in between 25% and 75% or high for above 75%. The 95% confidence interval (95% CI) excluding the null was considered significant or conclusive while 95% CI including the null was considered inconclusive. P-values lower than alpha (< .05) were interpreted as conclusive.

## 3. Results

### 3.1. Search result

**Figure 1.**
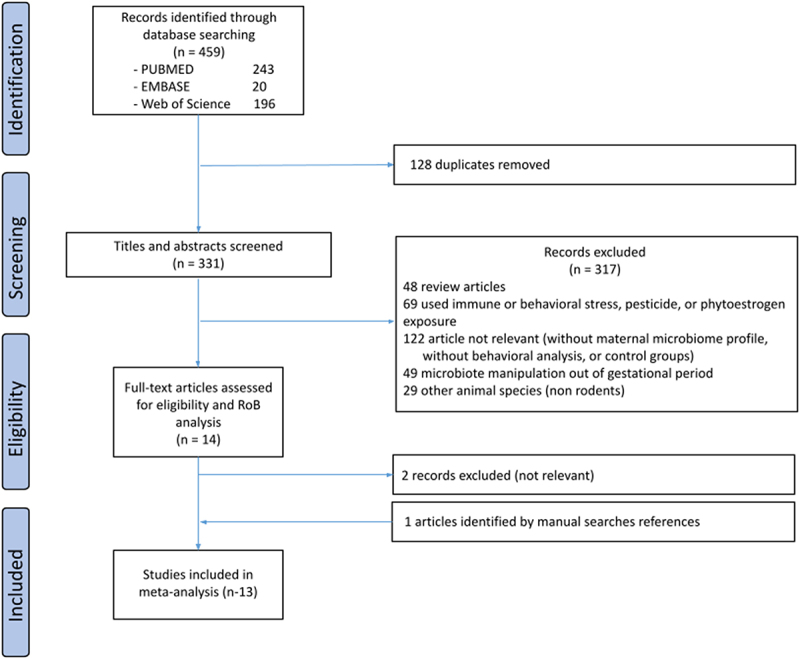
PRISMA flow diagram of the study.

From a total of 459 records identified in the database-searching strategy, 331 had the title and abstract screened after removing 128 duplicates (Figure 1). Fourteen articles fulfilled the eligibility criteria. After a full text examination, the two articles were excluded as they did not match the inclusion criteria.^36,37^ One eligible article found by a manual search was included.^38^ A total of 13 publications and 21 studies, reporting at least 1129 animals, were included in this review: Afroz et al., 2021;^39^ Bruce-Keller et al., 2017;^20^ Buffington et al., 2016;^40^ Champagne-Jorgensen et al., 2020;^41^ Hill et al., 2021;^42^ Lebovitz et al., 2019;^11^ Lyu et al., 2021;^28^ Sanguinetti et al., 2019;^43^ Tochitani et al., 2016;^27^ Vuong et al., 2020;^44^ Xiao et al., 2020;^45^ Yu et al., 2020;^46^ Leclercq et al., 2017.^38^

### 3.2. Description of the eligible articles

The characteristics of the selected articles are presented in Table 1. Most of the articles included in the present work used C57BL/6N (*N* = 9)^11,20,27,28,39,40,42,44,46^ mice as animal models. However, other rodent models were used, such as BALB/c (*N* = 2)^38,41^ and B6129SF2/J (*N* = 1) mice^43^ and Sprague-Dawley rats (*N* = 1).^45^

**Table 1.** Characteristics of the articles included in the present study.

Reference (author and year)	Behavioral paradigm	Experimental manipulation	Animal	Mean age (weeks)	Sex	Sample size	Behavior assessments
Lebovitz, 2019	Sociability	Antibiotic	C57BL/6N	3	ND	86	Three Chamber
Bruce-Keller, 2017		Fecal Transplantation	C57BL/6N	10	M; F	60	Ultrasonic vocalization
Tochitani, 2016		Antibiotic	C57BL/6N	12	M	29	Social interaction
Buffington., 2016		High-fat diet	C57BL/6N	10	M	32	Social Interaction
Jorgensen, 2019		Antibiotic	BALB/c	6	M; F	59	Three Chamber
Leclercq, 2017		Antibiotic	BALB/c	6	M; F	51	Three Chamber
Afroz, 2021		High-salt diet	C57BL/6N	9	M; F	52	Three Chamber
Bruce-Keller, 2017	OCD	Fecal Transplantation	C57BL/6N	10	M; F	60	Marble burying
Buffington., 2016		High-fat diet	C57BL/6N	10	M	32	Marble burying
Afroz, 2021		High-salt diet	C57BL/6N	9	M; F	52	Marble burying
Lyu, 2021		Silver nanoparticle	C57BL/6N	11	ND	23	Elevated plus maze
Vuong, 2020	Schizophrenia	Antibiotic	C57BL/6N	8	ND	70	Prepulse inhibition
Bruce-Keller, 2017	Anxiety	Fecal transplantation	C57BL/6N	10	M; F	60	Open field
Tochitani, 2016		Antibiotic	C57BL/6N	12	M	79	Open field
Buffington, 2016		High-fat diet	C57BL/6N	10	M	32	Open field
Jorgensen, 2019		Antibiotic	BALB/c	6	M; F	59	Elevated plus maze
Yu, 2020		Low-fiber diet	C57BL/6N	6	ND	34	Elevated plus maze
Lyu, 2021		Silver nanoparticle	C57BL/6N	11	ND	23	Elevated plus maze
Hill, 2021		Antibiotic	C57BL/6N	7	M; F	20	Open field
Leclercq, 2017		Antibiotic	BALB/c	6	M; F	51	Elevated plus maze
Bruce-Keller, 2017	Depression	Fecal transplantation	C57BL/6N	10	M; F	60	Sucrose preference
Yu., 2020	Memory	Low-fiber diet	C57BL/6N	6	ND	34	Barnes Maze
Sanguinetti, 2019		High-fat diet	B6129SF2/J	14	ND	45	Y maze
Lyu, 2021		Silver nanoparticle	C57BL/6N	11	ND	23	Barnes Maze
Xiao, 2020		Lead acetate	Sprague-Dawley	8	M	20	Morris Water Maze

OCD: obsessive-compulsive disorder; ND: non-defined; M: male; F: female.

Although most studies have split males and females into independent groups,^11,20,27,28,38–42,45^ some have not differentiated between the sexes.^43,46^ In this case, the animals were considered to have non-defined sex. As defined in the inclusion criteria, only animals tested from 21 days onwards were included in this meta-analysis. Thus, the animals underwent behavioral tests between an average of 3 and 14 weeks.

**Figure 2.**
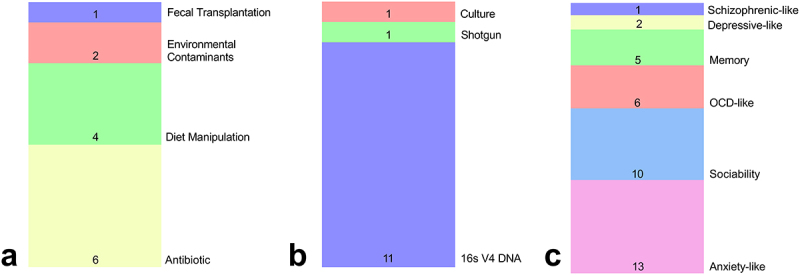
Descriptive analysis of the articles included in the present study. (a) The methodology used to manipulate the maternal gestational microbiota. (b) The method used to characterize the maternal gestational microbiota. (c) Behavioral paradigm analyzed.

The majority of included articles used antibiotics^11,27,38,41,44,46^ or diet manipulation^39,40,43,46^ as the experimental approach for maternal microbiota perturbation, followed by environmental contaminant by silver^28^ and lead^45^ and fecal transplantation^20^ (Figure 2a). Manipulations in the different selected articles that occurred during pregnancy and cold also included the breastfeeding period, encompassing the perinatal period. The 16s V4 DNA sequencing method for microbiome characterization was used for 11 articles,^11,20,27,28,34–44^ one used shotgun metagenomic,^28^ and one used bacterial culture^11^ (Figure 2b).

The eligible articles used the following outcome measures of behavioral analyses: (1) Open field: inner zone locomotor activity, time spent in the inner zone, or the number of entries in the inner zone; (2) Elevated plus maze (EPM): open arm exploration time (OAE), head dipping; (3) Three Chamber: sociability (score), time spent with social novelty, time in social chamber; (4) Ultrasonic vocalization (UV): vocalizing time; (5) Reciprocal social interaction (RSI): interaction frequency; (6) Barnes Maze (BM): latency to find the target; (7) Y maze (YM): alternation triplets; (8) Morris water maze (MWM): distance traveled in the target quadrant; (9) Marble burying (MB): number of buried marbles; (10) Prepulse inhibition test (PPI): prepulse inhibition; (11) Sucrose preference test (SP): volume consumption.

Thirteen independent groups of animals from the selected articles evaluated anxiety-related behavior, through Open Field^20,27,40,42^ or Elevated Plus Maze tests;^28,38,41,46^ ten accessed social deficits behaviors using Three Chamber,^11,38,39,41^ Ultrasonic Vocalization.^20^ or Reciprocal Social Interaction tests;^27,40^ six evaluated obsessive-compulsive behavior using Marble Burying test^20,39,40^ or Stereotyped Self-grooming;^28^ five surveyed memory through Barnes Maze.^28,46^ Y Maze,^43^ or Morris Water Maze;^45^ two assessed Depressive-like behavior applying Sucrose Preference test;^20^ and one investigated Schizophrenia using prepulse Inhibition test44 (Figure 2c).

### 3.3. Assessment of study quality

The Systematic Review Center for Laboratory Animal Experimentation (SYRCLE) risk of bias (RoB) tool was used to assess the risk of bias in the included animal studies. The risk of bias was low for most of the studies, as described in Supplementary Table S2.

### 3.4. Global meta-analysis: CESs of maternal enteric microbiome disruption on offspring behavioral outcomes

**Figure 3.**
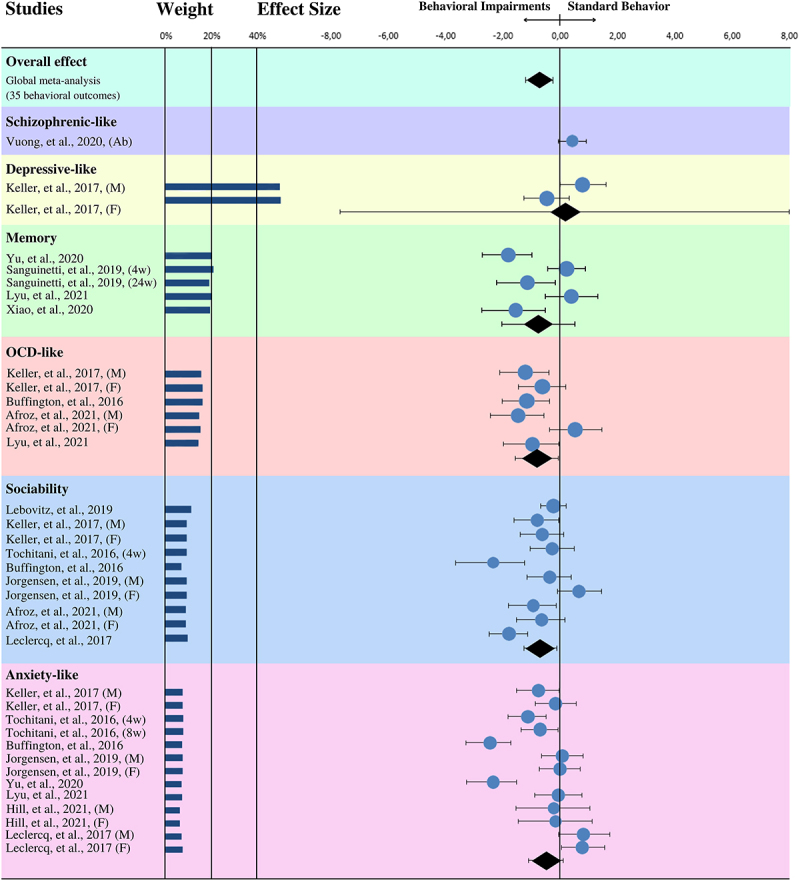
Forest plot graph of the effects of MMD on offspring behavior. M: males; F: females; Ab: Antibiotic; Gf: Germ-free. Only independent groups from the same study were used.

The analysis combined of all thirty-five behavior outcomes revealed a significant effect size (SMD = −0.51, 95% CI = −0.79 to −0.22, *p* < .001) indicating that MMD provokes behavioral impairments in the adult offspring (Figure 3). According to Hedges’g criteria, the effect size for the preset collection of data was moderate and associated with high heterogeneity (T2 = 0.54, I2 = 79.85%).

### 3.5. Stratified meta-analysis: CESs of maternal enteric microbiome disruption on offspring memory, and social, anxiety-like, depressive-like, schizophrenic-type, and obsessive-compulsive behaviors

The estimated CES in the meta-analysis stratified by each outcome reveals that MMD is significatively associated with reduction of sociability behavior (SMD = −0.63, 95% CI = −1.18 to − 0.07, *p* = 0.011, T2 = 0.30, I2 = 76.11%), and obsessive-compulsive-like behavior (SMD = −0.68, 95% CI = −0.01 to −1.36, *p* = 0.009, T2 = 0.25, I2 = 62.82%). According to Hedges’g criteria, the effect size for all stratified behaviors was moderate and associated with moderate heterogeneity (Figure 3).

The CESs were moderate and not significant statistically in the studies investigating anxiety-like behavior (SMD = −0.45, 95% CI = −1.07 to 0.17, *p* = 0.113, T2 = 0.83, I2 = 84.42%) and memory (SMD = −0.67, 95% CI = −1.85 to 0.50, *p* = 0.111, T2 = 0.79, I2 = 83.41%), and very small in depressive-like behaviors, also not significant statistically (SMD = 0.16, 95% CI = −7.16 to 7.46, *p* = 0.784, T2 = 0.53, I2 = 80.09%), both with high heterogeneity. As only one experimental group for schizophrenia-like behavior was found in the literature that fulfilled our inclusion criteria, it was not possible to perform a meta-analysis for this model (Figure 3).

## 4. Discussion

In the last decade, the maternal microbiota has received remarkable attention due to its interference with brain development, resulting in long-lasting effects on the offspring.^17,45^ According to the developmental origins of health and disease hypothesis.^46^ during the perinatal period, the development and maturation of several body regulatory systems, such as immune, endocrine, and neural systems, occurs with particular susceptibility to environmental factors.^47–49^ However, there is still a limited number of clinical studies investigating the role of the maternal enteric microbiome in infant neurodevelopment. Moreover, so far as we know, no study has directly investigated the effects of maternal dysbiosis on offspring’s behavior. Most protocols have used the GF animals or antibiotic, pre-, or probiotic treatments for microbiota manipulation. The present meta-analysis investigated whether the disturbance of maternal enteric microbiota would be associated with behavioral abnormalities manifested by the offspring during adult life. We access this hypothesis by reviewing thirteen eligible, from a set of 459 rodent preclinical studies, identified using a search strategy to determine offspring’s behavioral outcomes generated in MMD protocol.

One of the eligibility criteria requests the confirmation of disturbance of maternal enteric microbiota by at least one validated method. Only one article used growth culture media characterize the microbiota profile, while the other twelve used culture-independent methods. Among them, one study used the shotgun method, and eleven characterized the maternal microbiota by using the 16s metagenomics method.^50^ The metagenomic methods allow the characterization of a greater number of microorganisms and the processing of significant amounts of data than culture-dependent methods.^51^ The cost decrease observed in recent decades, especially to 16s metagenomics, explains the exponential number of studies in the field. Although the search strategy included other species as non-human primates, only studies with rodents matched all the criteria. Nine in thirteen studies used mice of the C57BL/6N strain as rodent models, three used other mouse strains (BALB/c and B6129SF2/J), and only one used rats (Sprague-Dawley).

This variability in species/strain may have contributed to the heterogeneity of the meta-analysis. Even though most studies have used GF compared to SPF animals, or protocols for microbiota recolonization of GF animals, the present meta-analysis did not include studies with GF protocols because this condition has been associated with several behavioral and neurodevelopmental abnormalities, and they have not been considered clinically relevant models because its weakness in causality.^52^

In the present meta-analysis, the combined effects for behavioral outcomes to animal models of major depression disorder, memory, obsessive-compulsive disorder, sociability impairments, and generalized anxiety reveal that MMD might negatively impact the expression of offspring’s behavior during adult life. The meta-analysis stratified for sociability outcomes confirmed a significant impact of MMD on the offspring’s behavior. This finding corroborates other studies showing that maternal microbiota might influence the social behavior of the offspring, being associated with increasing the risk of neurodevelopmental disorders including ASD.^18,53,54^ For instance, Bruce-Keller et al. demonstrated in 2017 that microbiota transplantation from animals submitted to the High Fat Diet (HFD) to pregnant females decreased the sociability of offspring in both males and females.^20^ Although this study did not investigate the neural mechanism, it suggested that a healthy maternal microbiota is important for the offspring`s social behavioral development. Lastly, Afroz et al.^39^ also observed that a high-salt diet during pregnancy reduced the maternal Lactobacillus population, which was related to lower sociability in the offspring, both in males and females.

Social impairments were also observed in studies that used small doses of antibiotics as models of MMD, an approach close to what is observed clinically. Jorgensen and colleagues demonstrated that even low levels of penicillin exposure can lead to social impairments only in male rodents. These behavioral alterations were correlated with brain AVPR1A, AVPR1B, and OXTR altered expression, and decreased balance of splenic FOXP3+ regulatory T cells.^38,41^ Prenatal penicillin exposure also led to distinct microbiota compositions clustered differently by sex.^41^ His team continued Leclercq et al.'s work, which in 2017 had already shown that small doses of penicillin during pregnancy and the perinatal period caused deficits in the animal's sociability.^38^ Regardless of gender, and an increased AVPR1b expression in the prefrontal cortex of both sexes, but not in the hippocampus. This gene encodes a receptor for vasopressin, important for arginine-vasopressin signaling, and is related to the aggressive behavior of offspring.^55^ The group also observed a systemic increase in pro-inflammatory cytokines, such as interleukin (IL)-6, IL-10, and CXCL15, in the animals’ prefrontal cortex, but without systemic changes.

After that, different studies analyzed brain impairments that could explain the effects of MMD, causing social disabilities in offspring. As observed by Lebovitz et al., one of the mechanisms by which a model of antibiotic-based bacterial depletion in maternal microbiota impairs the proper offspring neurobehavioral development is due to an accentuated prefrontal cortex microglial expression of Cx3cr1, a chemokine receptor for neuron-derived Cx3cl1 (fractalkine). This signaling pathway involves premature senescence of microglia and dysfunctional remodeling of synapses, leading to deficits in the social preference in males and females during the three-chamber test, which can be rescued by Lactobacillus murinus HU-1 or Cx3cr1 Knockout11.

Buffington and colleagues also explored the role of the Limosilactobacillus genus on mechanisms of social deficits induced by dysbiosis40. The group observed that pups of females that submitted to MMD showed social deficits related to lower long-term potentiation (LTP) in the ventral tegmental area (VTA) in dopaminergic neurons during social stimuli, and has a reduction in hypothalamic oxytocinergic neurons when compared to the pups generated in female control. Treating these pups with L. reuteri restores the number of hypothalamic oxytocinergic neurons, which reestablish LTP in the VTA and, consequently, improves the sociability of the animals.

Finally, the study conducted by Tochitani et al. did not observe social impairments in male mice.^27^ In that work, behavioral analysis was performed on the offspring of females submitted to MMD during pregnancy at four and eight weeks of life. However, the authors report that animals exhibit locomotor impairments at week four, which could be a confounding factor for the sociability test. Also, social behavior was not evaluated at week eight, when the animal no longer showed locomotor impairments. Taken together, these studies accumulate evidence that MMD modulates social behavior in the offspring and highlight the role of the Lactobacillus, Lacticaseibacillus, and Limosilactobacillus genus in sustaining a healthy microbiota for sociability in adult life.^11,20,27,38–41^

The CES stratified analysis also demonstrated that MMD increases the stereotypical behavior of the offspring. Stereotypical behaviors are symptoms of different disorders such as OCD, ASD^56^ and SZ,^57^,characterized by repetitive, functionless motor behavior.^57^ Stereotyped behavior seems to be related mainly to excessive dopaminergic activity in the basal ganglia,^58^ which is influenced by the gut microbiota. One of the gold standard models for studying this behavior in mice, with great validity and easy implementation, is the MB test.^59^ All three studies performed the test on males found that MMD elicits stereotyped behaviors in the offspring.^20,39,40^ Of these, only two also performed in females,^20,39^ which did not show significant differences from the control group. In addition to the MB test, another way to analyze stereotyped behaviors is through the quantification of unnecessary and exacerbated compulsive behaviors in mice, such as excessive grooming,^28^ increased chewing of non-nutritive kaolin clay,^60^ and excessive head-dipping behaviors.^60^ In the study by Lyu and collaborators, mice that underwent MMD through exposure to AgnNP spent more time engaged in stereotypic head-dipping behaviors compared to controls.^28^ Together these results suggest that MMD might have a sex-dependent effect for offspring`s stereotyped behaviors, corroborating the neurodevelopmental hypothesis for ASD.^61^

Regarding the other behaviors analyzed, it has already been demonstrated that colonization of healthy mice with fecal microbiota provided by donor mice subjected to stress protocols is sufficient to reproduce the stress-related behavior, including anxiety and depressive-like behaviors, in the recipient mice.^62^ In addition, Schmidt et al. have shown that FMT from healthy adult rats prevented both gut dysbiosis and anxiety-like behaviors development in those animals suffering spinal cord injury.^63^ Moreover, previous studies also have shown that adult GF animals exhibit a constitutive reduction of anxiety-related behaviors compared to SPF animals.^64^ This anxiolytic-like response of GF animals also occurs when they are exposed to stress,^65^ and it is reversed when mice receive microbiota of healthy SPF animals via FMT.^65^ Although all these pieces of evidence suggest that gut microbiota participants in anxiety-like responses, we found a marginal combined size effect of the MMD on offspring anxiety-like behavior in the present data set. However, we apply a meta-analysis for adult mice undergoing maternal enteric dysbiosis, a protocol different from the results above. Moreover, several animal models that explore anxiety-related behavior are susceptible to other environmental stress.^66^ Thus, studies aimed to assess the effects of MDD on offspring behavior could have been contaminated by confounding factors.

The same occurs with the CES for memory-related behaviors. D’Amato and coworkers have shown that transplantation of the microbiota from aged mice to adult mice affects spatial learning and memory via modulation of hippocampal synaptic plasticity.^67^ A sharp decrease in bacteria associated with the production of short-chain fatty acids (SCFAs) (Lachnospiraceae, Faecalibaculum, and Ruminococcaceae) was observed in the transplanted mice. Also, using transplantation techniques, the microbiota of a rodent model of Alzheimer’s disease for C57BL/6 mice decreased neurogenesis in the adult hippocampus and Brain-Derived Neurotrophic Factor (BDNF) expression, and increased p21 expression, leading to memory impairment.^68^ However, the results of stratified CES in the present study showed no effect of MMD on offspring memory, suggesting that these changes observed in adult animals, at least for the present collection of data, may not be related to MMD. Regarding behaviors related to depression and schizophrenia, the number of results obtained in the present data collection does not permit conclusions so far, and further studies are needed.

GBA is one of the most complex communication systems evolving immunological, endocrine, metabolic, and neural pathways.^69–75^ However, the mechanism whereby environmental factors during the perinatal-phase compromise the GBA and cause negative consequences to neural development remains to be elucidated.^76–78^

Dietary habits have been shown a consistent and reproducible factor in shaping the composition of the gut microbiome,^79^ leading to impairments in memory, exploration, and social behavior in adulthood in rodents.^40,43^ Different diets provide different nutrients that can select the composition of the microbiota, as shown in clinical^80^ and preclinical^81^ studies. In addition, various components of the maternal diet play beneficial roles in mental health depending on the gut microbiome.^82^ For example, inulin is a soluble fiber that cannot be digested by the body,^83^ but is metabolized by enteric microbiota, leading to the production of SCFAs.^84^ Several studies have reported that diet-induced MMD, e.g., inadequate dietary fiber or excessive consumption of high-fat or high-salt foods, disrupt the gut microbiota of the offspring^85^ and also modify the production of maternal gut metabolites that can cross the placenta and the embryo blood–brain barrier (BBB).^86^ Both have been associated with neurodegenerative diseases and behavioral disorders.^87^ In addition, transplantation of the HFD gut microbiota into female mice before breeding can disrupt social behavior in males, but not in females and offspring.^20^ This study adds to evidence that behavioral impairments induced by HFD may be vertically transmitted to the offspring through the microbiota in a sex-specific manner.

Another way to trigger MMD applied in the studies included in the present meta-analysis was the consumption of environmental contaminants, such as silver and lead. These substances are widely used by the food and pharmaceutical industries because of their beneficial antimicrobial and antifungal properties.^88^ However, chronic exposure to environmental pollutants has been linked to the development of neurodegenerative diseases, as these substances can accumulate in various organs, such as the brain and gut, with harmful effects. Moreover, due to their antimicrobial activity and accumulation in the gut, these materials can alter the microbiota when ingested, depending on the dose and format of the nanoparticles.^28^ In two papers presented here,^28,45^ environmental contaminants were found in association with MMD. The contaminant induced the reduction in resident hippocampal microglial cells and stereotyped behavior in the offspring.^28^ Memory impairment was also reported to be associated with morphological abnormalities in hippocampal dendritic spines,^45^ which could be reversed with a multispecies probiotic containing genus Bifidobacterium, Lactobacillus, Lacticaseibacillus, Limosilactobacillus, and Streptococcus.^45^ Together, these findings reinforce the role of environmental contaminants in MMD and its consequences to neurodevelopmental and mental health.

Antibiotic administration, in turn, has been pointed out as the most useful method for MMD induction generating a GF-like phenotype. These drugs may influence neurodevelopment by (1) altering the maternal gestational microbiota;^89^ (2) changing the profile of microorganisms passed to the offspring;^90^ (3) changing the absorption of nutrients by the mother, which may affect the microbiota and milk composition;^91^ (4) causing hyperactivation of the HPA axis by altering the maternal microbiota and impairing maternal nurturing behavioral in the postnatal period, which is a stress factor for the offspring.^27^ Yet, a common criticism regarding the use of antibiotics to deplete the microbiota in animal models is the lack of similarity with what actually occurs in clinics, which use significantly lower doses.^38^

Furthermore, different combinations and concentrations of antibiotics are used in the literature and, despite generally impacting the most abundant phyla such as Firmicutes and Bacteroidetes, this diversity causes different changes at lower taxonomic levels.^92^ In addition, clinical evidence has already shown that the individual’s microbiota before the start of antibiotic treatment is one of the main determining factors for the disturbances that will be caused in the microorganisms populations.^93^ Together, the combination of antibiotics used and the profile of the healthy microbiota of the animals used in each study might contribute to the heterogeneity observed between the different studies.

In the context of the neuro-immune system, a body of evidence suggests that inflammatory unbalance during pregnancy might facilitate neurodevelopmental psychiatric illness. Elevated levels of gestational maternal pro-inflammatory markers including C reactive protein (CRP) and pro-inflammatory cytokines such as IL-6, IL-1, and IFN-gamma have been associated with ASD,^94^ SZ,^95^ Parkinson^96^ and Alzheimer disease.^97–102^ Inflammatory environments seem to be related to a brain region-specific microglial activation, such as prefrontal cortex, hippocampus, and amygdala, with elevated expression of microglial senescence genes (such as Trp^53^ and I1β),^11^ inflammatory mediators (e.g. Cx3cr1),^11^ and reduced oxytocin signaling,^40^ which are correlated with dysfunctional modeling of synapses and behavior. Finally, evidence from our group and others^103^ has shown a remarkable effect on hippocampal biomarkers of synaptic plasticity when the dysbiosis is provoked during the weaning period (data not published). Therefore, the immune system has been considered a crucial for maintaining hippocampal neurogenesis, social behavior, and cognitive functions.^104,105^ In particular, for neurodevelopment, the gut maternal enteric microbiota has emerged as a crucial player in the immune system balance and maturation. For instance, in a model of reversible colonization of germ-free mice during gestation, the microbial shapes the neonatal immune system even before birth through molecular signals derived from the microbiota of the mother.^3^ Otherwise, maternal microbiota was important to mature intestinal innate immune cells and to alter intestinal gene expression profiles in the offspring.^3,106^ A clinical study with a cohort of 1074 newborn infants revealed that maternal gut microbiota might conditionate the composition of child immune cells.^107^ Infants clustered by Dialister, Escherichia, and Ruminococcus present lower proportion of granulocytes, and higher proportion of both central naïve CD4 T cells (CD4+/CD45RA+/CD31−) and naïve regulatory T cells (Treg) (CD4+/CD45RA+/FoxP3low). However, the meaning of this last finding for neurodevelopment remains to be elucidated. Therefore, the effect of MMD may have multiple pathways facilitate this bidirectional communication between the maternal gut microbiota and the offspring brain, including direct and humoral interactions of microbes or their metabolites with intrinsic and extrinsic neurons,^108–110^ lymphoid organs-derived soluble inflammatory mediators,^111^ and/or translocation of gut microbial products, such as tryptophan metabolites or SCFAs, into the brain circulation.^112–114^

Preclinical evidence is further consolidated regarding the neural pathways of enteric dysbiosis on behavior. For example, Sgritta et al.^4^ demonstrated that L. reuteri treatment reverses social deficits in a model of autism, including in GF animals, via activation of afferent fibers from the vagus nerve, an important nerve involved in the interaction between the periphery and CNS, for more details see.^115^ This group observed that L. reuteri treatment induces vagal signals to the hypothalamus . paraventricular nuclei (PVN), stimulating oxytocin production via neurons projecting to the VTA, restoring mechanisms of synaptic potentiation in this region.^4^ These findings link the adverse effects of maternal diet- and antibiotic-induced disruption of gut dysbiosis and neurobehavioral impairment of offspring.^20^

In addition, gut-brain communication also involves the production of hormones, neurotransmitters, and metabolites by enteric microbiota.^116^ These signaling molecules have local^117^ and systemic^118^ effects, and some of them can cross the BBB and act directly on the central nervous system. Regarding the impact of the maternal gestational microbiota on offspring neurodevelopment, preclinical^103^ and clinical^53^ studies in recent decades have focussed primarily on the production of maternal microbial metabolites capable of crossing the placenta and the embryo BBB,^119^ such as SCFAs. These small organic monocarboxylic acid molecules are the main products of microbial anaerobic fermentation of indigestible polysaccharides, such as dietary fiber, e.g., inulin, and resistant starch in the colon.^120^ Locally, the effects of SCFAs range from maintaining intestinal barrier integrity, mucus production, and protection against inflammation to reducing the risk of colorectal cancer.^106^ However, a fraction of these molecules also reach the systemic circulation, and they are capable of crossing both the placenta^119^ and the BBB,^119^ which is rich in SCFA transporters.^108^ In the brain, SCFAs regulate BBB integrity and function,^121^ promote microglial maturation,^122^ reduce neuroinflammation in models of LPS administration^123^ and ischemic stroke^124^, and affect neuronal function by modulating levels of neurotransmitters and neurotrophic factors.^108^ It is therefore speculated that SCFAs play a central role in both microbiota-gut-brain crosstalk^108^ and communication between the maternal microbiota and the embryo,^125^ consequently influencing neurodevelopment^46^ and behavior^126^ in the offspring.

Finally, it has been shown that some of the physiological impairments caused by altered gut microbiota may be antagonized, especially if treatment is given in the early stages of neurodevelopment, by (1) oral supplementation with specific probiotics (L. murinus HU-1 and L. reuteri);^11,40^ (2) co-housing with animals with a healthy microbiota;^40^ (3) treatment with metabolites from the maternal microbiota (e.g., butyrate, trimethylamine N-oxide, and imidazole propionate)^44,46^ and hormones (e.g., oxytocin);^40^ (4) or even targeting inflammatory components in the brain (e.g., Cx3cr1).^11^ Such manipulations may serve as future therapeutic approaches in an area that needs more research. The available meta-analysis suggests that maternal gut dysbiosis influences offspring behavior, which in turn is related to individual mental health and susceptibility to psychiatric disorders.

The principal limitation of the studies included in this meta-analysis is the lack of consistency in the standardization of protocols utilized among the different laboratories leading to low reproducibility. Many variables result in conflicting outcomes, including sexual dimorphism, behavioral tests, time and type of maternal dysbiosis utilized, assays to determine the microbiota, species, strain, and age of rodents, dose, and type of antibiotic that the animals were exposed to generate gut dysbiosis. Among these factors, the behavioral differences observed in sexual dimorphism were considered relevant given that, although many studies were performed utilizing male and female rodents,^11,20,28,38,39,41,42,44^ those that investigated males and females individually observed that the male was more sensitive to maternal microbiota-induced behavioral alteration than the females.^20,39,41,42^ Sexual dimorphism has already been reported for the social behavior parading in a study that used female offspring in the social interaction test.^127^ Even in humans, sexual dimorphism may influence the behavior or neurological outcomes linked to enteric microbiota. For instance, a study that evaluated the alpha diversity in six-week-old babies revealed that the higher diversity may be associated with internalizing (anxiety and depression) behavior only in boys, but not among girls, evaluated at age of three years old.^128^ Therefore, despite the high heterogeneity, the present study shows by the current scientific literature the importance of the maternal microbiota in the social behavior of the offspring. Further meta-analyses discriminating behavior outcomes for male and female animals might better clarify the sexual dimorphism associated with MMD.

Therefore, the present work demonstrates the importance of standardizing experimental paradigms in this new area of study so that the large amount of data generated can be analyzed more clearly. It confirms, from the current collection of references, which different environmental disturbances in the maternal enteric microbiota during the gestational period, such as antibiotic administration,^11,41^ diet,^40^ and environmental contaminants,^20,28,45^ can affect the behavior in the offspring during adult life.

## Conclusions

Our meta-analysis shows that maternal gut microbiota alterations during pregnancy generate changes in sociability and stereotypic behaviors related to psychiatric disorders in the offspring. The potential long-lasting behavioral disturbances of MMD in memory and mood disorders remain to be better investigated.

## Supplementary Material

Supplemental Material
