# Supplementary material for: Maternal microbiome disturbance induces deficits in the offspring’s behaviors: a systematic review and meta-analysis
Source: Gut Microbes. 2023 Jul 3;15(1):2226282. doi: 10.1080/19490976.2023.2226282 (PMC10321199; doi:10.1080/19490976.2023.2226282)
Supplement: Supplemental Material [file KGMI_A_2226282_SM2594.zip › Supplementary_Table_1.docx]

**Search Strategy**

Medline, Embase, and Web of Science were the database selected. The selection of keywords was based on the different denominations of mice, offspring, maternal, behavior, and microbiota (see Table 1). The access occurs at 11/03/2021.

**Supplementary table 1: Systematic review in specialized literature**

| Review # | Terms | Hits  PubMed | Hits  Embase | Hits Web  of Science |
| --- | --- | --- | --- | --- |
| 1 | (rodentia OR mice OR mus OR mouse OR murine OR rats OR rat OR murine OR muridae OR rodentia OR rodent OR rodents) | 3,772,913 | 4,432,844 | 3,661,051 |
| 2 | (Offspring OR infant OR adulthood OR progeny OR descendant) | 1,551,346 | 1,136,521 | 701,081 |
| 3 | (Maternal OR Gestational OR Pregnancy OR Prenatal OR Development OR  Neurodevelopment OR Preterm OR Mother OR perinatal OR pregnant OR germination OR parturiency OR childbearing) | 6,586,890 | 5,854,754 | 8,613,353 |
| 4 | (Behavior OR forced swimming test OR tail suspension test OR learned helplessness OR novelty suppressed feeding test OR sucrose spray test OR sucrose preference OR social default test OR elevated plus maze test OR vogel conflict test OR open field test OR light dark box OR elevated zero maze OR three chamber social interaction test OR three chamber social interaction test OR free social interaction OR ultrasonic vocalization OR water maze test OR novel object recognition test OR t test or marble burying OR prepulse inhibition OR depression OR anxiety OR MDD OR GAD OR major depression OR major depressive OR schizophrenia OR autism OR autism spectrum disorder OR panic OR obsessive compulsive disorder OR social | 5,571,458 | 252,509 | 5,376,076 |

|  | disorder OR social phobia OR mental health OR mental disorders) |  |  |  |
| --- | --- | --- | --- | --- |
| 5 | (Microbiota OR Microbiome OR Microbe OR Enteric microbiota OR Intestinal microbiota OR Gut microbiota) | 157,906 | 79,765 | 191,021 |
| 6 | #1 and #2 and #3 and #4 and #5 | 243 | 20 | 196 |
